# Formation and Application of Starch–Polyphenol Complexes: Influencing Factors and Rapid Screening Based on Chemometrics

**DOI:** 10.3390/foods13101557

**Published:** 2024-05-16

**Authors:** Yingying Wu, Yanan Liu, Yuanqiang Jia, Huijuan Zhang, Feiyue Ren

**Affiliations:** 1 Beijing Advanced Innovation Center for Food Nutrition and Human Health, Beijing Engineering, School of Food and Health, Beijing Technology & Business University (BTBU), Beijing 100048, China; 2130021027@st.btbu.edu.cn (Y.W.); 2230201031@st.btbu.edu.cn (Y.L.); 2330201004@st.btbu.edu.cn (Y.J.); zhanghuijuan@th.btbu.edu.cn (H.Z.); 2 Technology Research Center of Food Additives, School of Food and Health, Beijing Technology & Business University (BTBU), Beijing 100048, China

**Keywords:** complex formation, complex application, physicochemical property, digestibility

## Abstract

Understanding the nuanced interplay between plant polyphenols and starch could have significant implications. For example, it could lead to the development of tailor-made starches for specific applications, from bakinag and brewing to pharmaceuticals and bioplastics. In addition, this knowledge could contribute to the formulation of functional foods with lower glycemic indexes or improved nutrient delivery. Variations in the complexes can be attributed to differences in molecular weight, structure, and even the content of the polyphenols. In addition, the unique structural characteristics of starches, such as amylose/amylopectin ratio and crystalline density, also contribute to the observed effects. Processing conditions and methods will always alter the formation of complexes. As the type of starch/polyphenol can have a significant impact on the formation of the complex, the selection of suitable botanical sources of starch/polyphenols has become a focus. Spectroscopy coupled with chemometrics is a convenient and accurate method for rapidly identifying starches/polyphenols and screening for the desired botanical source. Understanding these relationships is crucial for optimizing starch-based systems in various applications, from food technology to pharmaceutical formulations.

## 1. Introduction

Starch is a pivotal raw material in the food industry, influencing both the sensory and nutritional attributes of products such as noodles and starch-based items. Comprising α-D-glucose units connected via glycosidic bonds, starch is a biopolymer. Structurally, it can be categorized into two main types: amylose and amylopectin. Amylose is a linear molecule composed of D-glucopyranosyl units linked by α-(1-4)-glycosidic bonds. In contrast, amylopectin exhibits a highly branched structure, with both α-(1-4)-glycosidic and α-1,6 glycosidic linkages [1,2]. The widespread use of starch in food manufacturing can be attributed to its accessibility, affordability, nutritional richness, and ease of processing. However, it presents challenges, including susceptibility to aging, retrogradation, limited freeze–thaw stability, and suboptimal water retention. These drawbacks can compromise the quality and longevity of starch-based products. Some functional food components have been used to improve the physiochemistry and functional properties of starch [3]. It is evident from studies that the combination of these functional components and starch results in the modulation of sensory and taste properties of starch-based foods, as well as alterations in the digestion and absorption patterns of starch [4].

**Figure 1:**
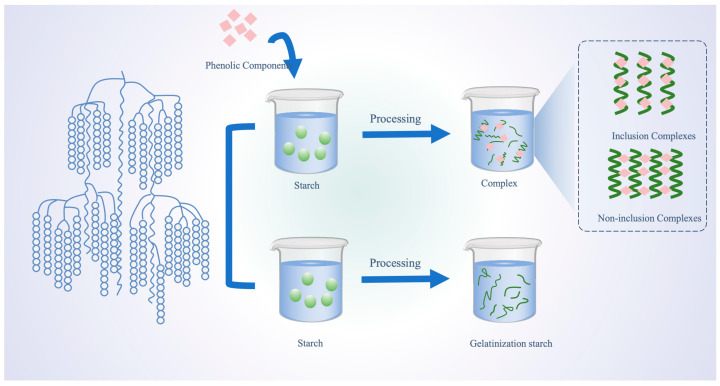
The formation of inclusion and non-inclusion starch–polyphenol complexes during processing.

**Figure 2:**
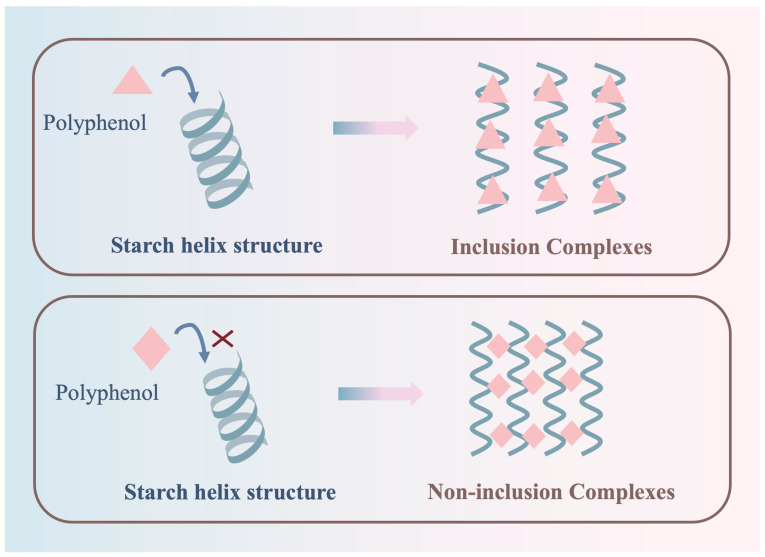
The difference in formation of inclusion and non-inclusion complexes.

Plant polyphenols are compounds prevalent in plants, characterized by multiple phenolic hydroxyl groups in their fundamental structure [5]. Acknowledged as potent bioactive entities, plant polyphenols boast numerous health benefits, including antioxidant, antibacterial, and antitumor properties [6,7]. Lately, the intricate molecular interactions between plant polyphenols and starch have garnered significant interest [3]. These polyphenols offer potential as bioactive ingredients in the crafting of functional starch-based foods. Polyphenols are known to form complexes with starch under external forces, with these complexes classified into two main categories: V-type inclusion complexes and non-inclusion complexes (Figure 1) [8,9,10]. The fundamental distinction between the two classes of complexes is their respective ability to enter the single-helix structure of starch (Figure 2) [11]. Upon entering the cavity of the starch helical structure, polyphenols can form a V-type inclusion complex with starch. In contrast, when polyphenols are unable to enter the helical structure, a different complex is formed, known as a non-inclusion complex [12]. Over time, valuable insights and methodologies regarding the molecular interplay of polyphenols with starch have been developed and refined. As science and technology advance, the chemical intricacies of these molecular interactions are becoming clearer. The scope of research has expanded beyond traditional food processing, encompassing chemical and biological dimensions, thus emerging as a focal point in food science and related fields [13,14]. Drawing extensively from research and the literature, this paper elucidates the molecular interactions between plant polyphenols and starch, shedding light on their reciprocal effects. The aim is to offer insights to optimize the utilization of plant polyphenols in starch processing, storage, and related areas.

## 2. Effects of Polyphenols on the Formation of Starch–Polyphenol Complexes

The effects of polyphenols on complex generation are not uniform but depend on multiple variables including: molecular size, the number of hydroxyl groups, and the spatial configuration of phenolic compounds. The interactions between plant polyphenols and starch result in diverse impacts on starch microstructure and its physiochemistry properties [15,16].

### 2.1. Catalogue of Polyphenols

Polyphenols are natural chemical substances that contain one or more aromatic rings with one or more hydroxyl groups in their structures [17]. Due to their polyhydroxy structures, polyphenol have the ability to form hydrogen bonds with the hydroxyl side chains of starch molecules. As plant secondary metabolites, they have a broad range of structural diversity, ranging from simple individual aromatic ring structures to complex condensed tannins. 

They can be generally categorized into phenolic acids, flavonoids, anthocyanins, and tannins, etc. [18]. Phenolic acids are classified into hydroxybenzoic acid, which is characterized by one carboxylic group, and hydroxycinnamic acid, which is characterized by a two-carbon skeleton (C_6_H_5_CHCHCOOH) with at least one replaceable hydrogen molecule that can be substituted by a hydroxyl group [19]. The components derived from hydroxybenzoic acid include *p*-hydroxybenzoic, gallic, protocatechuic, and vanillic acids. Similarly, hydroxycinnamic acid leads to *p*-hydroxycinnamic, *p*-coumaric, caffeic, and ferulic acids [18]. Flavonoids are derivatives of benzo-γ-pyrone that consist of a phenolic ring and a pyran ring [20]. According to the degree of oxidant of central heterocycle, flavonoids can be classified into various subclasses: flavonols, flavones, isoflavones, anthocyanidins, flavanones, flavanols, and chalcones [5]. Catechin (flavanol), rutin (flavone), kaempfeol (flavonol), naringin (flavanone), genistin (isoflavone), and apigenidin (anthocyandidin) are typical flavonoid antioxidants [20,21]. Tannins can be categorized into two types: condensed tannins (also known as proanthocyanidins) and hydrolysable tannins. Hydrolysable tannins have carbohydrates as a central core and hydroxyls esterified with phenolic groups, while condensed tannins are polymers of flavan-3-ols coupled by carbon–carbon bonds [18,22]. 

**Figure 3:**
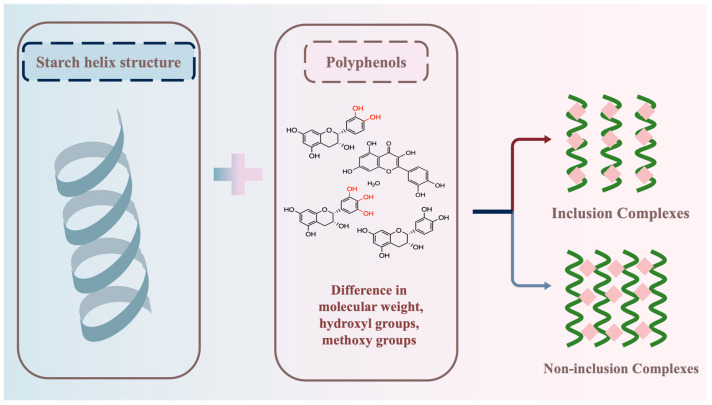
Effect of polyphenols on the formation of starch–polyphenol complexes.

Polyphenols can form complexes with starch through hydrogen bonding. However, due to the structural variability of polyphenols (such as molecular weight, hydroxyl groups, methoxy groups of polyphenols), there may be differences in the formation of starch–polyphenol complexes. The ability of polyphenols to combine with starch may vary, and the types of complexes formed by polyphenols and starch may also differ (Figure 3).

Both quercetin and rutin have been found to reduce the storage and lost modulus of Tartary buckwheat starch paste. However, rutin demonstrates a greater impact, possibly due to its unique molecular structure [23]. Rutin has an additional glucose and an additional rhamnose attached to its C ring, making it more polar than quercetin. This enhanced polarity and the presence of extra hydroxyl groups allow rutin to form more hydrogen bonds with starch molecules, thereby affecting the rheology to a greater extent. The impact of polyphenols on the rheological properties of starch is influenced by various factors such as the type and concentration of polyphenols, as well as the composition and source of the starch. The molecular structure of the polyphenols also plays a significant role, as evidenced by the difference in the effects of quercetin and rutin on wheat starch. This understanding is crucial for tailoring the rheological properties of starch for specific applications, especially in food and pharmaceutical industries where textural attributes are vital.

### 2.2. Molecular Weight of Polyphenols

The complexes were classified as either inclusion or non-inclusion complexes, which were influenced by whether or not the polyphenols entered the helical cavity of the starch [3,8,11]. The molecular weight of polyphenol played an important role in complex formation [11,14]. This is attributable to the varying steric hindrance and flexibility of polyphenols with varying molecular weights, which determines their fate in the context of starch helices, specifically whether the polyphenols are included in the helices or trapped between helices [24,25].

Polyphenols with a lower molecular weight have a lower spatial resistance, allowing them to more easily enter the helical cavity of the starch to form inclusion complexes [26,27]. For example, gallic acid, because of its smaller molecular size, functions as a ‘bottle stopper’ through hydrogen bonding and hydrophobic interactions in the hydrophobic cavity of starch. The starch–polyphenol complex formed by protocatechuic acid has a higher relative crystallinity than ellagic acid, naringin and tannic acid, which have a higher molecular weight [27].

Inclusion starch–polyphenol complexes can be classified as V_6_, V_7_, and V_8_ complexes, with the numbers 6, 7, and 8 representing the number of glucose subunits per turn of the starch single helix [28]. It is conceivable that the V_6_, V_7_, and V_8_ complexes exhibit differences in their structural and physicochemical properties. For instance, it has been demonstrated that the V_6_ complex displays a more ordered starch–polyphenol structure and is more thermally stable [29,30]. The type of complex formed appears to be influenced by the molecular weight of the polyphenol. The smaller molecular weight polyphenol such as caffeic acid (Mw 180.16) formed V_6_-type complexes [16]. Resveratrol (Mw 228.24) interacted with maize starch and formed V_6_- and V_7_-mixed complexes [31]. As for chlorogenic acid, with Mw of 354.31, formed V_7_-amylose helix with lotus seed starch [32].

In contrast, bulky polyphenols with greater molecular weight encounter difficulty when entering the helix crave of starch and probably cause starch aggregation [33]. For example, tannic acid, with its larger molecular structure and high number of hydroxyl groups, was found to be a significant promoter of the binding and aggregation of starch molecules [14,27]. Chen, Gao, He, Yu, and Zeng [27] simulated the mechanism of complex formation between tannin aid and starch. It was found that due to steric hindrances, the tight wrapping of the short chain glucose is prevented despite the tannins having enough hydroxyl groups. In addition, the large polyphenol molecule acts as a bridge, connecting the starch and causing them to aggregate [34]. The hydrogen bonding between the hydroxyl groups of tannic acid and amylose leads to an increase in starch crystallinity and a decrease in starch digestibility [14].

### 2.3. Phenolic Hydroxyl Groups

An important feature of polyphenols is their abundance of hydroxyl groups, which not only confers them notable antioxidant properties and bioactivity but is also an important reason why polyphenols are able to interact with starch. Hydrogen bonds are formed between the hydroxyl group of the polyphenol and the starch, resulting in the formation of complexes [35,36]. The hydrogen bonding maintains the stability of polyphenols in the hydrophobic cavity of starch in inclusion complexes. In non-inclusion complexes, polyphenol binding to the starch chain via hydrogen bonding as a bridge and aggregate the surrounding starch molecules with itself as the core. Noticeably, the hydroxyl groups in polyphenols may affect their binding affinity to starch, and as such can influence the structural, physiochemical, and functional properties of starch [37,38]. The ability to form hydrogen bonds can be conferred by the electron transfer capacity and reactivity of the hydroxyl group [39,40].

#### 2.3.1. Number of Hydroxyl Groups

The quantity of hydroxyl groups in polyphenols is a crucial factor in the formation of these complexes, leading to alterations in the physicochemical properties and structural characteristics of starch. The increase in hydrogen bonds hinders the intermolecular interaction of starch chains, thereby altering the structure of starch [15,41]. For instance, epigallocatechin gallate (0.0175 mol OH groups/g) has a higher number of hydroxyl groups per gram compared to caffeic acid (0.0166 mol OH groups/g) and quercetin (0.0167 mol OH groups/g). As a result, when forming a complex with maize starch, EGCG has a greater potential for OH groups and the ability to form hydrogen bonds with starch [35]. A study by Yu, Zhu, Li, Zhong, Huang, and Chen [41] indicates that caffeic acid, which has two hydroxyl groups on the benzene ring, binds most strongly to corn starch, followed by ferulic acid, with 1 hydroxyl group, and cinnamic acid, with no hydroxyl groups. 

Binding with polyphenols may disturb the dense porous network structure of native starch gels, causing them to become loosely structured with irregular fragments. The higher number of hydroxyl groups in the polyphenols intensifies the disruption [42]. Furthermore, the presence of more hydroxyl groups on the phenolic component facilitates its interaction with starch by occupying more binding sites on the starch chain, thereby modifying the short-range ordered structures of starch [42,43]. The change in starch structure determines the extent to which the physiochemical properties of the starch are altered, such as rheology, gelatinization, and retrogradation [42,43,44].

#### 2.3.2. Spatial Distribution of Phenolic Groups

The distribution of hydroxyl groups is also important for their combination with starch molecules [27]. The spatial arrangement of hydroxyl groups influences the electron cloud, thereby altering the surface electrostatic potential of the phenolic entities, which in turn affects their affinity and reactivity with starch [39]. A study by Fan et al. [45] found that the ortho-hydroxyl structure enhances the interaction between polyphenols and starch more than the meta-hydroxyl structure. Complexes prepared with ortho-hydroxyl structures of polyphenols, such as protocatechuic acid and caffeic acid, exhibited the higher relative crystallinity of starch. Additionally, polyphenols with ortho-hydroxyl structures demonstrated better encapsulation and loading efficiency [45].

### 2.4. Methoxy Group

In addition to the hydroxyl group, methoxy, another functional group on the benzene ring, affects the interaction between polyphenols and starch. Notably, the presence of methoxy group increases the hydrophobic interaction between polyphenols and starch with starch [43]. The increase in electron density is closely related to hydrophobic interactions, possibly caused by the presence of methoxy groups on the benzene ring [46]. A study by Fan, Yao, Chen, Ma, Wen, Li, Wang, and Sun [45] found that a higher concentration of methoxy groups promotes the interaction between polyphenols and starch. The complexes formed by ferulic acid with methoxy groups had a higher ordered crystalline structure, resulting in the highest encapsulation and loading efficiency.

### 2.5. Concentration of Polyphenols

**Figure 4:**
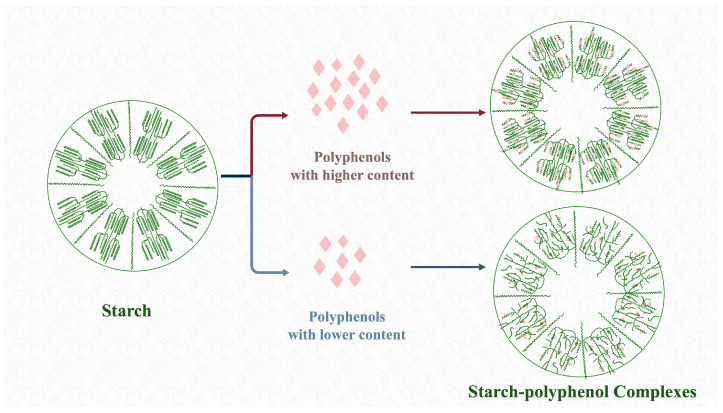
Effect of concentrate of polyphenols on the formation of starch–polyphenol complexes.

With an increase in concentrate, the hydrogen bonding in the system of starch and polyphenols also increases, which certainly promotes the formation of complexes (Figure 4) [47,48]. However, there has been no significant positive effect on the formation of the long-range ordered structure of starch with increasing polyphenol concentration, although the short-range ordered structure was changed [47].

It is worth noting that polyphenol solutions are acidic due to their rich hydroxyl groups. At lower concentrations, polyphenols have minimal influence on the pH of the starch–water system. In this scenario, the primary molecular interactions between polyphenols and starch involve hydrogen bonding, van der Waals forces, and hydrophobic interactions. Due to the limited hydroxyl groups available in the phenolic substances, only minor modifications to the semi-crystalline structure of starch granules are observed [4,49]. When the concentration of polyphenols is elevated, significant changes in pH occur, resulting in a more acidic environment for starch. Increased acidity leads to stronger hydrogen bonding between starch granules, thereby inhibiting the interactions among free starch chains. For example, higher concentrations of tea polyphenols were observed to reduce the final viscosity of rice starch paste, attributed to the disruption in the orderly arrangement of starch chains [50]. Polymeric proanthocyanidins with a lower content (1%) reduced the viscoelasticity of the high-amylose maize–starch system, inhibited amylose rearrangement, and enhanced its fluidity. However, excessive proanthocyanidins restrained the interaction between proanthocyanidins and amylose [51].

The concentration of polyphenols is important for the physicochemical and functional properties of complexes. This varying influence of polyphenol concentration has implications for food formulation and processing, allowing for a degree of customization in the final product’s texture, stability, and nutritional profile. Incremental additions of ferulic acid led to reductions in both onset and final gelatinization temperatures. While a modest decrease in gelatinization enthalpy was observed with 5%, 10%, and 15% ferulic acid concentrations, a significant drop was evident at a 20% concentration [38]. Additionally, the inhibitory effect of polyphenols on starch retrogradation is also concentration-dependent. Increasing concentrations of tea polyphenols have been found to progressively reduce the retrogradation enthalpy and rate of rice starch [52]. The increase in chlorogenic acid content promoted the interaction between polyphenols and starch, disrupted the binding and aggregation between starch chains, resulting in the enhanced stability of the complex [9]. Studies employing rapid viscometer analyses and differential scanning calorimetry have indicated that as the concentration of tea polyphenols increases, both the gelatinization temperature and enthalpy of rice starch decrease [53].

As the concentration of Tartary buckwheat polyphenol increases (1–4%), the gelatinization enthalpy of Tartary buckwheat starch first increases and then decreases, while that of wheat starch gradually decreases [54].

In conclusion, an increase in the concentration of polyphenols facilitates the interaction between polyphenols and starch, leading to alterations in the physicochemical properties of starch. However, changes in polyphenol concentration do not seem to affect the type of complex formed. The molecular weight and substituent groups of polyphenols appear to influence the type of complex that is formed.

### 2.6. Polyphenols from Different Botanical Sources

Different botanical sources such as tea, fruits, vegetables, and cereals contribute to the variety of polyphenols available. When preparing starch–polyphenol complexes, researchers often opt to use naturally sourced extracts containing multiple monomeric phenolics instead of a single monomeric phenolic. This is because there may be a synergistic effect between multiple monomeric phenolics that promotes complex formation [34]. For instance, a study conducted by Lv, Li, Pan, Zhang, Jiang, Liu, Zhu, and Zhang [34] revealed that in comparison to the single compounds (epigallocatechin gallate, epicatechin gallate, epigallocatechin and epicatechin), the complexes prepared from tea polyphenols formed more hydrogen bonds and exhibited a higher crystallinity of starch.

#### 2.6.1. Tea Polyphenols

Tea polyphenols consisting mainly of catechins such as (−)-epigallocatechin gallate, (−)-epigallocatechin, (−)-epicatechin gallate and (−)-epicatechin were observed to retard the retrogradation of wheat starch [34,55].

Guo, Zhao, Chen, Chen, and Zheng [12] proposed a model for non-inclusion complexes between green tea phenols and starch. The green tea phenols act as molecular bridges that connect the outer side of starch chains through hydrogen bonding, thereby altering the crystallization, morphology, and particle size of lotus seed starch. 

Adding tea polyphenols disrupts the gel properties of starch. Specifically, the hydroxyl groups in these polyphenols hinder the interactions between starch molecules that are necessary for the formation of a gel network. Consequently, there is a weakening in the gel strength of the starch. This effect shows a dose-dependent relationship, increasing in intensity as the concentration of tea polyphenols rises [34]. Tea polyphenols influence the starch gel microstructure by enlarging pore sizes and enhancing surface roughness [54,56]. Additionally, green and black tea polyphenols also show differential effects on potato starch retrogradation, likely due to their distinct phenolic profiles arising from the level of fermentation [57]. The gelatinization parameters across rice, corn, and potato starches have been observed to substantially reduce after combined green tea polyphenols. The magnitude of these changes is influenced by the original gelatinization enthalpy and amylose content in these starches [58]. 

#### 2.6.2. Cereal Polyphenols

Cereals are a rich source of polyphenols. Phenolic acids such as ferulic acid, p-coumaric acid, and o-coumaric acid are commonly found in cereals [59]. The differing structures of polyphenols result in varying affinities for starch, which in turn gives rise to differences in the physicochemical properties of starch–polyphenol complexes.

For example, Tartary buckwheat polyphenols impact the initial and peak gelatinization temperatures of both Tartary buckwheat and wheat starch, but not their final gelatinization temperatures. The influence on gelatinization enthalpy also varies with the concentration of Tartary buckwheat polyphenols [54]. Purple red rice bran anthocyanins form inclusion complexes with rice starch through non-covalent bonding, which confers antioxidant properties to the starch [60]. Furthermore, they alter the structure of rice starch, reducing its digestibility.

The incorporation of buckwheat polyphenols causes a significant loss of transparency in starch pastes made from both wheat and buckwheat. This occurs because the polyphenols encourage the aggregation of starch molecules that are otherwise dispersed post-gelatinization. As these molecules clump together, they limit the passage of light, thereby reducing transparency [23,54].

## 3. Effect of Starch Properties on the Formation of Starch–Polyphenol Complexes

**Figure 5:**
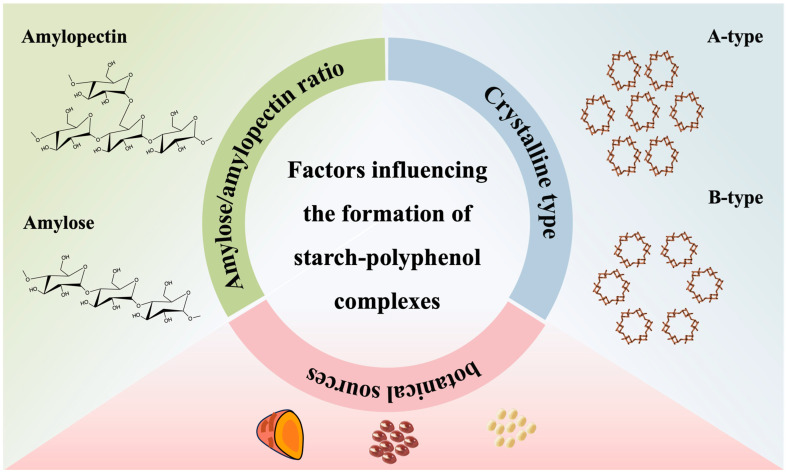
Effect of starch properties on the formation of starch–polyphenol complexes.

Starch extracted from various plants, such as maize, potato, and rice, has distinct structural characteristics that influence its interaction with polyphenols. The generation of complexes is affected by the reaction of the same type and concentration of polyphenols with different types of starch. Variations in starch properties, such as crystalline structure, particle size, and the ratio of amylose to amylopectin, play a crucial role in determining the physicochemical and functional properties of starch–polyphenol complexes. This is likely due to the varying binding affinities between polyphenols and starch with different properties (Figure 5).

### 3.1. Amylose/Amylopectin Ratio

Natural starch is composed of linear amylose (composed of α-(1-4)-linked D-glucose units) and highly branched amylopectin (composed of α-(1-4)-linked D-glucose backbone with α-(1-6)-linked branches) [61]. The amylose-to-amylopectin ratio varies with botanical origin and affects the structural and morphological properties of starch, including molecular weight, granule morphology, and the proportion of ordered crystalline to amorphous domains [62].

Long chains of amylose undergo intramolecular interactions, forming helix cavities with hydrophobic properties that provide the basis for polyphenol attachment [63]. Some studies have indicated that amylopectin may have difficulties interacting with polyphenols [35]. However, in fact both amylose and amylopectin have the potential to form complexes [28]. It has also been shown that the helical structure in amylopectin occurs in the long-branched chain, although the ability to produce this structure is limited [12,64].

The ratio of amylose to amylopectin in starch affects binding affinities with polyphenols. The increased proportion of amylose promotes the ordering of the polyphenol–amylose complex crystallites, resulting in highly crystalline degrees of amylose [11]. For example, Ma et al. [65] revealed that corn starch with a higher amylose content exhibits a higher complexing index with apigenin through hydrogen bonding. In contrast, there is no significant intermolecular interaction between apigenin and amylopectin. Similarly, a study by Liu et al. [66] that compared normal maize starch and amylopectin maize starch, highly amylose maize starch was found to have stronger binding ability with oligomeric procyanidins, resulting in complexes with more resistant starch.

### 3.2. Different Starch Crystalline Structures from Different Botanical Backgrounds

Starches can be classified into four categories based on their origin: cereal, tuber, legume, and other types [67]. Starches from different plant sources vary considerably in terms of crystalline structure, particle size, and morphological properties, which can affect their processing characteristics. Potato starch has a compact and condensed outer layer that helps to maintain the structural integrity of the granules under high hydrostatic pressure. In contrast, both pea starch and corn starch fully gelatinize and expand [68].

Depending on the double helix arrangement of starch, there are three types of starch: A, B, and C crystalline starch. A-type structures have tightly packed glucose helices, while B-type structures have less dense packing, allowing water molecules to enter between the branches. C-type structures are a combination of the A- and B-types [69]. Polyphenols have varying effects on the structure and physiochemistry properties of starch from different botanical sources. Compared to corn starch and pea starch, potato starch has a better anthocyanin binding rate [68].

The study found that green tea polyphenols significantly reduced the enthalpy of retrogradation and the retrogradation rate of rice starch, maize starch, and potato starch after gelatinization. However, green tea polyphenols were more effective in inhibiting the retrogradation process of rice starch and maize starch than that of potato starch [55,58].

#### 3.2.1. A-Type Cereal Starch

As an effective tool for starch crystal structure analysis, X-ray diffraction (XRD) is wildly used. The strong peaks around 15°, 17°, 18°, and 23° (2θ) can be identified in A-type starch. Cereal starches, such as wheat starch, Tartary buckwheat, corn starch, rice starch, etc., can be identified as A-type starches, with strong peaks around 15°, 17°, 18°, and 23° (2θ). After being combined with polyphenols, the crystalline structure of starch is altered, resulting in a new, ordered structure. Combined with Blue honeysuckle extracts leading the crystalline structure of corn starch change from the native A-type crystals into V-type crystals [70]. The characteristic peaks of the V-type crystals, observed at 12.8 and 19.9 (2θ), often indicate the formation of an inclusion complex [70]. The proanthocyanidins entered the helical cavities of rice starch and formed A + V-type starch–polyphenol complex [71]. However, it should be noted that V-type crystals are not observed in non-inclusive complexes [72].

The physicochemical and structure properties of wheat starch are also changed. Wheat starch interacts with tea products through hydrogen bonding, which interferes with the rearrangement of starch chains and delays retrogradation during storage [34]. Moreover, it has been reported that the formation of complexes reduces the digestibility of cereal starches such as rice, maize, and Tartary buckwheat [73,74,75].

#### 3.2.2. B-Type Tubers Starch

According to their XRD pattern, strong peaks around 17° and 22° (2θ) can be identified in B-type starch. Potato starch has a typical B-type crystalline structure, which has been studied extensively [76]. Polyphenols interfere with the rearrangement of starch and the formation of the double-helix structure, and this change may lead to changes in starch crystal type and crystallinity. Research has indicated that the formation of V-type complexes between potato starch and dandelion flavonoids through non-covalent bonds leads to a reduction in the starch digestion rate coefficient (*k*), signifying a decrease in the rate of starch enzymatic hydrolysis [48]. Additionally, the terminal digestibility (*C*∞) decreases, suggesting a lower degree of the enzymatic hydrolysis of starch [48]. Similarly, when combined with polymeric proanthocyanidins with different degrees of polymerization, the B-type diffraction peak disappears and V-type peaks are generated [77]. However, a study by Xu, Dai, Chen, He, Shuai, Liu, and Li [77] found that when subjected to ultrasound, potato starch can form non-V-type complexes with rose polyphenols.

This process does not alter the crystalline type of potato starch, but it does effectively decrease its viscosity and increase the amount of resistant starch.

The physicochemical characteristics of tuber starch, such as viscosity, retrogradation, and digestibility, may be affected by interactions between starch and polyphenols. Proanthocyanidins restrain the retrogradation of potato starch, including short-term and long-term retrogradation [77]. Grape seed proanthocyanidins can also form a complex with potato starch, altering the physicochemical properties of the starch [78]. The decrease in the final viscosity and hardness and the increase in the thermal stability of starch can be observed in the potato starch–grape seed proanthocyanidins complex [79].

#### 3.2.3. C-Type Starch

With well-defined typical diffraction peaks at 2θ of 15°, 17°, and 23°, lotus seed starch is a typical C-type starch. Its crystalline form would change if inclusion complexes are formed. When polyphenols enter the helical cavities of amylose, starch crystallites can transform into a V-type [80,81,82].

Via hydrogen bonding, tea polyphenol generates V-type complex with the amylose and the linear side chain of amylopectin of potato starch under conditions of high-pressure homogenization [79]. Similarly, C-type pea starch could form the V-type crystalline with ferulic acid. Not all complex formation is accompanied by a change in crystalline type. It has also been shown that non-inclusion complexes can be generated without a new crystalline type, maintaining the original type of the starch [83]. The physiochemistry properties of C-type starch change after combination with polyphenols via non-covalent binding. The digestibility of lotus seed starch decreases and the long-term retrogradation of lotus seed starch is restrained by interaction with chlorogenic acid [84,85]. Strong H-bonds between green tea polyphenols and starch lead to an increase in the swelling power and solubility of starch [83]. 

### 3.3. Various Botanical Sources of Starch

#### 3.3.1. Maize Starch

Maize starch is a high-yielding source of starch. However, its physicochemical properties make its application in the food industry limited. In order to alter the physicochemical properties of maize starch, polyphenols have been employed to bind to starch. Polyphenols, due to their structural feature of containing hydroxyl groups, are able to interact with the hydroxyl groups on the starch chains in maize starch, preventing the formation of hydrogen bonding between the starch double-chain bonds [27]. This results in a change in the arrangement of the starch chains, which subsequently affects the microstructure and physicochemical properties of starch.

To illustrate, quinoa polyphenols and maize starch can form inclusion or non-inclusion complexes, which retard starch gelatinization and reduce digestion rate [74]. Similarly, the binding of ferulic acid modifies the swelling, pasting, gelatinization, retrogradation, and gel texture of corn starch [38]. In another study, Zhang et al. [86] finds that the combination of quercetin with maize starch resulted in the dissolution of the crystalline structure of the starch, leading to the formation of new crystalline regions. This process significantly reduced the digestibility of maize starch and increased the content of resistant starch.

#### 3.3.2. Rice Starch

Rice is a cereal grain that is widely used as a source of energy. Through hydrogen bonding, polyphenols can interact with starch chains and bind [87,88]. Previous studies have demonstrated the potential of polyphenols to alter the structure and physicochemical properties of rice starch. 

In a study conducted by Han, Zhang, Zhang, Huang, Jia, Huang, and Liu [15], it was observed that the presence of polyphenols (ferulic acid, gallic acid, and quercetin) led to a looser, more porous gel matrix of gelatinized rice starch. This phenomenon was hypothesized to be due to the polyphenols enhancing the water-holding properties of the rice starch. Another study, by Igoumenidis, Zoumpoulakis, and Karathanos [87], demonstrated that caffeic acid inhibits the reassociation of starch chains, thereby retarding the retrogradation of rice starch. A similar finding was observed in a study by Wu, Chen, Li, and Li [88], where tea polyphenols exhibited a similar effect on rice starch. Furthermore, the addition of appropriate concentrations of gallic acid resulted in alterations to the short-range molecular order and aggregation of rice starch, which in turn led to an increase in resistant starch content and a decrease in pGI values [89].

## 4. Effect of Processing on the Formation of Starch–Polyphenol Complexes

### 4.1. The pH Condition of Starch and Polyphenols

Starch and polyphenol affinity were significantly modulated by reaction pH. The structure of starch and polyphenols undergoes changes depending on the pH. For example, anthocyanins are more stable under acidic conditions, while alkaline conditions may cause their degradation [90]. Therefore, under acidic conditions, inclusion complexes of anthocyanins and Amylopectin nanoparticles (APNPs) bind at a higher rate. As the pH increases from 1 to 3, the binding rate also increases, resulting in the formation of inclusion complexes. However, at higher pH levels, the binding rate of inclusion complexes decreases [90]. Similar results were observed in a study on the preparation of complexes of cyanidin-3-O-glucoside and potato starch [91]. The optimum preparation condition was found to be pH 3, and the main force sustaining both was electrostatic interaction. 

It appears that lower pH conditions are more favorable for polyphenol and starch interactions.

However, a contrasting perspective is presented in Li et al. [92], which suggests that an increase in pH is conducive to the formation of complexes. The authors observed that the dominant interaction force between starch and polyphenols underwent a gradual transition from electrostatic interactions to hydrogen bonding as the pH increased. Additionally, it was found that the binding rate and amount of binding of both polyphenols and starch increased with increasing pH.

### 4.2. The Processing Methods of Starch–Polyphenol Complexes

In recent years, various processing methods, including microwave, ultrasound, extrusion, and cold plasma treatment, have been used to prepare starch–polyphenol complexes. Processing techniques can be used to produce additional complexes beyond the limited number achievable through physical mixing [15,79,93]. Compared to physical mixing, these methods facilitate the interaction between starch and polyphenols by disrupting the double-helix structure of starch, which exposes more binding sites for polyphenols and promotes the formation of complexes.

The degree of degradation to starch and the promotion of the complex varies according to the technology used. Therefore, the use of various processing methods can effectively regulate the formation of complex types. For instance, the co-gelation of starch with tannic acid results in the formation of mostly non-inclusion complexes; however, it is possible for mixtures of the two to produce inclusion complexes [8]. In a study by Zhou et al. [94], it was found that gelatinization and ultrasonic treatment lead to the dissolution of amylose and the destruction of the double-helix structure. This alteration in starch structure facilitates the formation of a V-type complex between Tartary buckwheat starch and flavonoids. 

On the other hand, high hydrostatic pressure treatment causes a gentle gelatinization that allows the complex to retain the original starch crystal form.

#### 4.2.1. Microwave Processing

Microwave processing is an efficient processing method that utilizes the vibration of polar groups to convert electromagnetic energy into thermal energy for the rapid heating of food products [95]. Microwave processing transfers heat uniformly into the granules through internal heating, accelerating the disruption of starch helices, releasing more starch chains and exposing more binding sites capable of binding polyphenols, which promotes the formation of complexes [9]. Following microwave processing, the starch combines with the polyphenol and formed V-type crystalline, while it forms a C-type crystalline structure following physical mixing [83].

These alterations in starch structure result in changes to the physicochemical properties of starch. At a microwave power of 250 W or 300 W, the binding of chlorogenic acid and starch is more effectively promoted, thus inhibiting the short-term retrogradation of starch [96]. Furthermore, microwave processing results in a reduction in starch digestibility, as it facilitates the formation of chlorogenic acid–lotus starch V-type complexes [84].

#### 4.2.2. Ultrasound Processing

The role of ultrasonication in promoting complex formation has been demonstrated in several studies. The acoustic cavitation of ultrasound generates a significant shearing force and turbulence, which leads to the disruption of starch granules and an increase in effective collisions between polyphenols and starch, thereby promoting the formation of starch–polyphenol complexes [97]. This phenomenon has been corroborated by numerous studies.

For instance, a study conducted by Raza et al. [98] demonstrated the effectiveness of ultrasound treatment in facilitating the exposure of starch chains, as well as enhancing the interaction of tannins with these chains. This, in turn, results in a notable enhancement of the thermal stability and rheological characteristics of the complex, accompanied by a notable rise in the content of resistant starch. Similar findings were observed in a study by He et al. [99], which demonstrated that the canistel seed starch formed a non-conclusion complex with quercetin via H-bonding and van der Waals interactions was facilitated by the action of ultrasound. The aforementioned structural alterations have the effect of enhancing the exothermic stability of the starch complex.

#### 4.2.3. Extrusion Processing

Extrusion plays a relevant role in modifying the interaction between starch and polyphenols. Extrusion encompasses a series of steps including fusion mixing, cooking, kneading, shearing, and molding. During this process, the starch is subjected to heating and expansion, the double helix is unwound and the hydroxyl groups of amylose become exposed, facilitating the interaction of polyphenols with starch chains [100]. This conclusion has been corroborated by multiple studies. For example, following the extrusion process, chlorogenic acid forms a V- type complex with rice flour, resulting in a notable reduction in starch digestibility [100]. Zheng, Tian, Ogawa, Kong, Chen, Liu, and Ye [37] observed that, following extrusion, grape seed proanthocyanidins and starch formed complexes through hydrogen bonding, resulting in altered pasting properties, thermal characteristic, and a significant reduction in starch digestibility. Furthermore, the authors postulated that this facilitating effect was related to the increase in the porosity of the rice by extrusion, which increased the contact between polyphenols and starch [37].

## 5. Polyphenol and Starch Diversity: Rapid Identification Based on Spectroscopy Techniques Coupled with Chemometrics

The formation of a complex can be fundamentally affected by the structural differences between polyphenols and starch. The amount of amylose in the starch and the concentration of polyphenols also play roles in the formation of complexes. Different starches or polyphenols are selected to form complexes depending on the intended purpose. Therefore, it is important to quickly select the desired source of starch or polyphenols from a wide variety of plant-based materials. Screening raw materials using a combination of spectroscopy and chemometrics can provide detailed information on food samples and efficiently screen desired samples for food evaluation.

### 5.1. Rapid Identification of Amylose Content in Starch

The amylose content affects the formation of complexes, resulting in the creation of complexes with unique physicochemical properties and digestive properties.

Vibrational spectroscopy has great potential for food characterization, particularly near-infrared (NIR), mid-infrared (MIR), and Raman spectroscopy. These methods are fast, efficient, and require no sample preparation, reducing time and reagent consumption. Additionally, they do not damage the sample. However, they often generate a large amount of data, which necessitates the use of auxiliary chemometric methods. Chemometric methods are efficient for processing data and constructing models. Generally, these methods are widely used in starch characterization.

#### 5.1.1. NIR Spectra

NIR spectra are an ideal technique for the determination of starch content [101], amylose content [102], starch viscosity [103], starch hydrolysis [104], etc. The C-H, N-H, and O-H bonds in starch vibrate upon interaction with NIR light, resulting in a characteristic spectrum. Data from NIR spectra can be analyzed by chemometric methods for the development of mathematical models for the rapid determination of amylose content.

A calibration model based on NIR spectra accomplishing a modified partial least square (MPLS) for brown rice amylose content was evaluated for 173 brown rice varieties [105]. Additionally, 29 samples were used for external validation. The calibration models had a high coefficient of determination and low error, with a standard error of cross-validation (SECV) of 1.52. A recent study by Xie et al. [106] applied an NIR spectroscopy technique to the concurrent determination of amylose and amylopectin in rice flour. The best model of MPLS has a high coefficient of determination (RSQ) of 0.928, and 0.912 for amylose and amylopectin content, respectively. Sampaio et al. [107] proposed an accurate quantification of the amylose content of rice via NIR spectra combined with chemometric tools. Compared to partial least squares (PLSs), interval PLSs (iPLSs), and moving windows PLS (MWPLS), synergy interval PLS (SiPLS) regression has the highest accuracy and lowest error with a correlation coefficient (R) of 0.94.

In general, NIR spectra coupled with chemometrics are highly accurate and reliable for quickly identifying the proportion of amylose in various starches. They facilitate the preparation of complexes by enabling the selection of starches with the appropriate proportion of amylose for processing.

#### 5.1.2. Raman Spectra

In addition to NIR spectra, Raman spectra can also be used for non-destructive diagnosis of starch characterization, such as predicting starch digestibility [108] and amylose content. Raman spectroscopy is a form of scattering spectroscopy that can identify characteristic chemical bonds or functional groups for the purpose of molecular structure analysis. Coupled with chemometrics [109], reliable prediction models for food composition can be constructed. Almeida et al. [110] developed a model that can be used for the rapid identification of amylose content using Raman spectroscopy and has been subjected to multivariate calibration regression using PLS. Moreover, the prediction error of the model was similar to that of the standard colorimetric method.

### 5.2. Phenolic Content

As previously stated, the phenolic content significantly influences the formation of complexes. Therefore, screening plant sources with higher polyphenol content is advantageous for complex preparation. Spectroscopic methods coupled with chemometrics provide a fast and cost-effective way to measure polyphenols with high processing capacity [111]. This method makes it possible to quickly discriminate between varieties that have a high content of polyphenols.

For instance, Rizvi et al. [112] developed NIR coupled with PLS regression to quantify the total phenolic content of *Citrullus colocynthis*. This regression model accurately determines polyphenol content in a non-destructive and efficient manner. Similarly, Fernández and Agosin [113] developed a method for rapidly quantifying red wine tannins using Fourier-transform mid-infrared (FT-MIR) and chemometrics. Resende et al. [114] used FT-IR and PLS regression to evaluate the content of Cyanidin-3-glucoside (C3G), ellagic acid (EA), and delphinidin-3-glucoside (D3G) in jaboticaba peel flours. The model proved to be highly accurate, and the measured contents were reasonably matching those of HPLC.

Chemometric analysis allows for the creation of a MIR spectroscopy database of phenolics. Mike analyzed the MIR spectral database using PCA and found that the spectral regions of 1755–1400 cm^−1^ and 1000–870 cm^−1^ could distinguish most of the phenolics [115]. For instance, methoxy can be found in the 1470 and 950 cm^−1^ bands, while flavonoids have multiple peaks in the region of 1650–1400 cm^−1^ and regions below 1200 cm^−1^.

Additionally, chemometric methods may be employed to optimize the extraction rate of polyphenols from plants. The extraction process of spent coffee grounds polyphenols was optimized by Ramón-Gonçalves et al. [116] using the response surface methodology (RSM) and the optimum conditions were found to be 15 min at 60 °C using 25% ethanol as the extraction solvent. Zheng et al. [117] utilized RSM with a Box–Behnken design (BBD) to optimize the extraction process of phenolic components from foxtail millet bran. Similarly, Pinto et al. [118] employed RSM with a central composite design (CCD) to optimize the extraction of phenolic antioxidants from chestnut shells.

Considering the synergistic effect, it may be more advantageous to use polyphenol extracts containing a mixture of phenolics when preparing complexes. The chemometric method optimizes extraction and allows for the rapid quantification of polyphenols. This expands the range of phenolics available for use in the preparation of starch–polyphenol complexes.

## 6. Application of Starch–Polyphenol Complexes in the Food Industry

### 6.1. Starch as an Encapsulation Agent

Polyphenols, although beneficial for health, are sensitive to factors such as high temperatures and oxidative stress, which can lead to their degradation during processing, storage, or digestion. Ensuring the bioavailability of polyphenols—that is, their absorption into the bloodstream—presents a significant challenge [6,45].

One strategy to address this issue is encapsulation, which can protect polyphenols from degradation and enhance their stability. For instance, the starch–genistein inclusion complex demonstrates remarkable stability under simulated gastrointestinal conditions [119]. Notably, the plasma concentration of genistein from this complex was twice as high as that from a physical mixture of starch and genistein, pointing to increased bioavailability.

Among various encapsulating materials, starch stands out for its availability, safety, affordability, and biocompatibility. Starch can encapsulate a broad spectrum of phenolic compounds such as catechins, anthocyanins, resveratrol, and tannic acid. This encapsulation occurs within cavities formed through intermolecular cross-linking post-gelatinization. Depending on their molecular compatibility, starch and polyphenols form either V-type or non-V-type complexes through hydrogen bonding and hydrophobic interactions [49]. These complexes offer dual benefits: first, they protect polyphenols from digestive degradation; second, they regulate the release of these compounds, optimizing their bioavailability [12]. In certain cases, encapsulation also improves the solubility of low-solubility phenolic compounds like quercetin and curcumin [120].

Utilizing modified starches can further elevate encapsulation efficacy. For instance, octenyl succinic anhydride-modified wax corn starch has demonstrated superior encapsulation and sequential release of epicatechin during digestion [121]. Similarly, acetylated starch nanoparticles improve curcumin’s stability and bioavailability [122], while 4-α-glucosyltransferase-modified rice starch enhances both curcumin’s solubility and bioavailability [123].

### 6.2. Starch–Polyphenol Complexes and Their Influence on Digestibility

Starch is categorized into three types based on its digestibility: rapidly digested starch, slowly digested starch, and resistant starch [124]. Digestibility is modulated by factors such as the presence of polyphenols. Studies have consistently demonstrated that polyphenols reduce the digestion rate of starch [89,125]. For example, maize starch exhibited reduced digestibility upon complexation with caffeic acid, and this effect was positively correlated with phenolic concentration [71]. In sum, both inclusion and non-inclusion complexes of polyphenols with starch lead to decreased starch digestibility, increasing the fraction of resistant starch.

#### 6.2.1. Polyphenol-Mediated Inhibition of Digestive Enzymes

Starch digestion is facilitated by enzymes like α-amylase and α-glucosidase, which produce shorter-chain carbohydrates such as maltose and α-limit dextrin that are further hydrolyzed to glucose [126,127]. Polyphenols, including flavonoids and phenolic acids, have been shown to inhibit these enzymes [128,129]. The inhibitory mechanisms involve competitive and non-competitive interactions where polyphenols either compete with starch for enzyme active sites or modulate enzyme functionality by binding to different regions [129,130]. The specific type of inhibition appears to depend on the chemical structure of the polyphenol.

#### 6.2.2. The Role of Starch Structure in the Starch–Polyphenol Complex

Digestibility studies have indicated that the starch–polyphenol inclusion complex is less susceptible to enzymatic digestion than a physical mixture of starch and polyphenols [11,73]. This decreased digestibility arises not only from the inhibitory effect of polyphenols on enzymes but also from the formation of V-type crystalline structures in the complex. These helical conformations are less accessible to α-amylase due to their size and rigidity [29,35,131]. Consequently, the starch–polyphenol complex exhibits increased resistance to enzymatic digestion, implicating its utility in enhancing resistant starch content.

### 6.3. Modifier of Food Quality 

The transformation of physiochemistry properties is crucial for many food and industrial applications where the properties of the starch are critical to the end product (Table 1). Water absorption, swelling, viscosity, and rheology are all important indicators of food quality in food production. Different foods have different requirements for these physical and chemical properties.

Starch regeneration is undesirable in the production and storage of products, as it can lead to poor product taste, starch caking, etc. Starch regeneration is caused by hydrophobic interactions and strong hydrogen bonding between starch molecular chains [133,134]. Numerous studies have shown that the interaction between plant polyphenols and starch can inhibit starch regeneration to a certain extent [25,43]. This is due to the fact that the highly reactive phenolic hydroxyl groups of phenolic compounds can form hydrogen bonds with the hydroxyl groups of amyloses, which prevents the entanglement of amylose molecules and inhibits the alignment of the polymer chains of amylose [51,85].

Flow and viscosity properties are key rheological characteristics of starch pastes which is important for food production. During the process of pasting, the chains of amylose molecules within the starch granules can move continuously due to their flexible structure and connect to the network structure generated by the intermolecular double-helix entanglement [135,136]. The interaction between plant polyphenols and starch can affect the fluidity of starch molecules and change the viscosity parameter of the reaction system, thereby affecting the rheological properties of starch [53,137].

The reduction in hardness is a result of the product’s improved quality due to the addition of polyphenols. As storage time increases, the product’s hardness gradually increases, which is caused by water loss [138]. The addition of polyphenols impedes the rearrangement of the starch’s more rigid double-helix structure, resulting in a decrease in product hardness. Therefore, polyphenols are highly resistant to the negative effects of water loss.

### 6.4. Application of Starch–Polyphenol Complexes in Starch Products

The attention paid to the incorporation of bioactive ingredients into popular foods has grown rapidly due to increased consumer health awareness together with a good acceptability of the products [139]. Polyphenols have been added to various products, including pasta and bread, to achieve the unique flavor and color of these starch products. The direct addition of polyphenols to starch products has improved the antioxidant properties of the final products. Furthermore, the addition of polyphenols has increased the content of resistant starch in starch products due to the unique structure of the complex.

The color, texture, and cooking properties of potato starch noodles were altered by protocatechuic acid, naringin, and tannic acid [77]. A study by Wang et al. [140] was conducted to produce extruded noodles with polyphenols and buckwheat starch. The polyphenols formed a V-type inclusion complex with the starch in the noodles, leading to a more controlled release of phenols and slower starch digestion. A study by Wang et al. [141] reveals that the addition of buckwheat phenol reduced the amount of reducing sugars released from noodles with buckwheat starch and phenolic extract. Additionally, most of the phenolic compounds were released during the gastric phase and effectively inhibited α-amylase activity.

Additionally, the anthocyanins extracted from black rice interact directly with starch in a bread matrix [142]. This interaction modifies the microstructures and physicochemical properties of starch, resulting in a dose-dependent decrease in starch digestibility.

## 7. Conclusions

Interactions between plant polyphenols and starch are influenced by a variety of factors, including their molecular structure, the spatial configuration of the polyphenols, and the botanical origin of the starch. These interactions have far-reaching effects on the physicochemical properties, processing behavior, and digestibility of starch-based foods. In addition, they can enhance the bioavailability and bioactivity of polyphenols, providing opportunities to optimize both starch digestibility and the benefits of polyphenols in foods. Although the ability of polyphenols to improve starch digestibility has been demonstrated, further in vivo studies are needed to better explain the antidigestion mechanism of the complex.

To promote complex formation, different processing methods are employed, including co-heating, microwave, and extrusion. Some innovative techniques, such as cold-plasma treatment, high-static pressure treatment, and high-pressure homogenization treatment, are also used for complex preparation, as they facilitate complex formation more efficiently with less energy consumption. Nevertheless, research on these technologies is still in its infancy and further research is needed. Further research is urgently needed to elucidate the optimal production technologies, clarify the mechanistic pathways, and identify their applications in functional foods.

Starch–polyphenol complexes have great potential for industrial applications, but there are only a limited number of studies investigating the practical applications of this complex. Consequently, there is a pressing need for further research to examine the potential of starch–polyphenol complexes in modifying the properties of actual food products. Such research can provide novel insights and solutions for the development of products in the food industry and other sectors, thereby facilitating the commercialization of starch–polyphenol complexes.

## Figures and Tables

**Table 1:** Effect of the formation of complexes of starch and phenolics on their physiochemistry properties.

Starch	Polyphenol	Complex Type	Characterization Techniques	Reference
Rice starch	Catechin	V-type	Resistant starch increased α-amylase inhibition	[132]
Lotus seed starch	Green tea phenolic	Non-inclusive complexes and V-type inclusion complex	Digestibility lowered	[79]
Proso millet starch	Proanthocyanidins	-	Relative crystallinity decreased Solubility and swelling power of starch increased Enthalpy value decreased Resistant starch increased	[47]
Potato starch	Dandelion flavonoids	-	Textural parameters decreased Moduli decreased Resistant starch increased Temperatures and enthalpy of gelatinization increased	[48]
Wheat starch	Tannic acid	Non-inclusive complexes and V-type inclusion complex	G′ and viscosity increased Digestibility slowed down	[8]
Rice starch	Proanthocyanidins from Chinese berry leaves	-	Enthalpy value decreased Thermal stability decreased Digestibility lowered	[71]

## Data Availability

No new data were created or analyzed in this study. Data sharing is not applicable to this article.
